# Pneumococcal puerperal mastitis in a lactating mother

**DOI:** 10.1099/acmi.0.000020

**Published:** 2019-04-24

**Authors:** T. Skalidis, A. Stamnidi, V. Syriopoulou, G. Kontopoulos, N. Legakis

**Affiliations:** 1 Central Laboratories, IASO Gynecology, Maternity and Pediatric Hospital, IASO Group Hospitals, Athens, Greece; 2 Medical School, National and Kapodistrian University of Athens, Athens, Greece; 3 IASO Gynecology, Maternity Hospital, Athens, Greece

**Keywords:** puerperal mastitis, pneumococcal infection, breast-feeding

## Abstract

**Introduction:**

A case of pneumococcal mastitis in a breast-feeding mother 6 months postpartum is described. Mastitis is usually caused by *Staphylococcus aureus *. A review of the literature from 1950 to March 2018 revealed only four other cases in which the causative organism was *Streptococcus pneumoniae *.

**Case presentation:**

The nursing mother presented with high fever and the four cardinal signs of inflammation of the left breast: calor, dolor, rubor, tumour. In milk culture *Streptococcus pneumoniae* was isolated in numbers exceeding 10^5^  c.f.u. ml^−1^ . The strain was of polysaccharide serotype 11 not included in Prevnar-13. Susceptibility testing showed full sensitivity to *β*-lactam antibiotics as well as to macrolides, lincosamides, vancomycin and tetracycline.

**Conclusion:**

*
Streptococcus pneumoniae* should be considered as a possible causative agent of puerperal mastitis.

## Case report

A 37-year-old woman who was breast-feeding her 6-month-old baby boy presented to our facility (IASO Gynecology, Maternity and Pediatric Hospital, Athens, Greece) with a 2-day history of diffuse headache, high fever (39–40 °C) and general malaise. Her previous medical history was unremarkable. Physical examination revealed an extremely tender left breast that was erythematous, indurated and warmer in the left lower lateral quadrant. A slight enlargement of the left axillary lymph nodes was also present. No pus collection could be detected by palpation and no purulent discharge was observed. However, mild periareolar pressure caused the expulsion of purulent milk from the left nipple.

The patient was subjected to an extensive laboratory evaluation but the results were unremarkable. The blood count was normal, except for the presence of a slight leukocyte left-shift with 83 % neutrophils (total white cell count of 9.2×10^3^ μl^−1^).

Culture of the breast secretions taken at the time of physical examination revealed heavy pure growth of *S. pneumoniae* (>10^5^ c.f.u. ml^−1^ of milk). The strain tested by Quellung reaction, using antisera provided by Staten Serum Institute (Copenhagen, Denmark), was of polysaccharide serotype 11. Antibiotic susceptibility testing was performed using Vitek-2. The strain proved to be susceptible to penicillin, cephalosporins, erythromycin, clindamycin, vancomycin and tetracycline. Prior to the milk culture results the patient was treated empirically with Cefaclor 500 mg, three times/day, she was discharged and advised to continue the same treatment for another 6 days.

The baby did not show any evidence of respiratory tract or any other infection prior to and during our patient’s illness. Nasopharyngeal culture of the baby was not performed as he was unavailable for culture sampling.

## Discussion

The incidence of puerperal mastitis ranges from 2.6 to 33 % [1]. The majority of cases of infectious mastitis are caused by *Staphylococcus aureus* followed by coagulase-negative staphylococci, group B streptococci, viridans streptococci and enterococci [2]. Pneumococcal mastitis is an extremely rare infection. There have been only four other case reports in the literature [3–6] (Table 1). *S. pneumoniae* isolates are usually associated with pneumonia, meningitis and bacteremia. However, there have been rare reports of pneumococcal skin and soft tissue infections most of which refer to patients with some degree of immunosuppression [7]. Our patient was an immunocompetent 37-year-old woman with no signs of any underlying disease. Although it has been stated that healthy breast-feeding women may harbour potentially pathogenic bacteria in their breast milk [2], the purulent material expelled from the nipple and the fact that pneumococci may cause breast abscess [8] prompted initiation of empiric antibiotic therapy, which was continued for a short while as it was relevant to the results of the antibiotic susceptibility testing.

**Table 1.** Cases of pneumococcal puerperal mastitis

Author	Age of patient (years)	Time from birth (months)	Localization of mastitis	Milk culture results	Culture result from the child
Present case	37	6	Left breast	*S.* *pneumoniae* serotype 11	Not tested
Miedzybrodzki and Miller [3]	35	8	Right breast	*S.* *pneumoniae* serotype 19A	Not tested
Wüst *et al*. [4]	38	9	Left breast	* S. pneumoniae* serotype 6B	* S. pneumoniae* serotype 6B
Kragsbjerg *et al*. [5]	38	4	Left breast	* S. pneumoniae *	* S. pneumoniae *
Hald and Schønheyder [6]	29	3	Right breast	* S. pneumoniae* serotype 19F (plus positive blood culture)	Not tested

Although the woman’s baby did not show any signs of respiratory tract infection and no culture from the baby’s nasopharynx was performed, it is highly likely that the mother was infected with *S. pneumoniae* from the baby’s nasopharyngeal secretions during breast-feeding, as it has been described in previous case reports [4, 5]. The baby was vaccinated at 2 and 4 months of age with Prevnar-13 (Wyeth, Collegeville, PA, USA), which contains capsular antigens for 13 *S*. *pneumoniae* serotypes (1, 3, 4, 5, 6A, 6B, 7F, 9V, 14, 18C, 19A, 19F, 23F) from the 91 described until now [9]. Thus, *S. pneumoniae* serotype 11 isolated from our patient is not part of the 13-valent PCV administered in Greece since 2010. Since the introduction of PCV-13 there has been growing concern for the development and spread of pneumococcal serotypes not included in the vaccine. Anecdotal information from the country suggest that serotype 11 *S*. *pneumoniae* is increasingly isolated from nasopharyngeal flora as well as from invasive infections.

### Conclusion

Our case report clearly demonstrates that a pneumococcal serotype not included in the vaccine may play a significant role in puerperal mastitis. In addition, a hint is provided that a serotype replacement phenomenon may occur in response to the introduction of conjugate vaccines. The replacement of vaccine-serotypes by others most probably arises as a consequence of selection for antigenic diversity imposed by the human immune system [10].

## References

[1] World Health Organization (2000). Mastitis: Causes and Management. WHO/FCH/CAH/00.13.

[2] Kvist LJ, Larsson B, Hall-Lord M, Steen A, Schalén C (2008). The role of bacteria in lactational mastitis and some considerations of the use of antibiotic treatment. Int Breastfeed J.

[3] Miedzybrodzki B, Miller M (2013). A lactating woman presenting with puerperal pneumococcal mastitis: a case report. J Med Case Rep.

[4] Wüst J, Rutsch M, Stocker S (1995). *Streptococcus pneumoniae* as an agent of mastitis. Eur J Clin Microbiol Infect Dis.

[5] Kragsbjerg P, Norén T, Söderquist B (1995). Deep soft-tissue infections caused by *Streptococcus*
*pneumoniae*. Eur J Clin Microbiol Infect Dis.

[6] Hald Vester S, Schønheyder HC (2018). *Streptococcus pneumoniae* as a cause of lactational mastitis: a case report. Clin Case Rep.

[7] Garcia-Lechuz JM, Cuevas O, Castellares C, Perez-Fernandez C, Cercenado E (2007). *Streptococcus pneumoniae* skin and soft tissue infections: characterization of causative strains and clinical illness. Eur J Clin Microbiol Infect Dis.

[8] Appalaraju B, Mathews AA, Bhaskaran AC, Arunachalam P (2011). Breast abscess caused by penicillin resistant *Pneumococci*. J Family Community Med.

[9] Reinert R, Jacobs MR, Kaplan SL (2010). Pneumococcal disease caused by serotype 19A: review of the literature and implications for future vaccine development. Vaccine.

[10] Spratt BG, Hanage WP, Bruegemann AB, Tuomanen El (2004). Evolutionary and population biology of *Streptococcus pneumoniae*. The Pneumococcus.

